# Supplementary material for: A Likelihood Approach for Real-Time Calibration of Stochastic Compartmental Epidemic Models
Source: PLoS Comput Biol. 2017 Jan 17;13(1):e1005257. doi: 10.1371/journal.pcbi.1005257 (PMC5240920; doi:10.1371/journal.pcbi.1005257)
Supplement: S1 File — (TAR.GZ) [file pcbi.1005257.s014.tar.gz › HSPH_Online-SI-Revision/MSS10/n10-extreme/n10-extreme_NEW2_table0.pdf]

1	newly 3 weeks spec	724.	631.49	{594., 669.}	12.7776	0.059951	newly 3 weeks cumul	3019.	2758.49	{2665., 2838.}	8.62902	0.0393263	newly 5 weeks spec	306.	263.73	{231., 300.}	14.402	0.0690913	newly 5 weeks cumul	3848.	3432.51	{3351., 3515.}	10.7976	0.0497447	newly 58 weeks cumul	4437.	3871.59	{3748., 4053.}	12.7431	0.0594351
2	newly 3 weeks spec	551.	477.98	{438., 521.}	13.2995	0.0629701	newly 3 weeks cumul	1831.	1671.95	{1568., 1769.}	8.68651	0.0399196	newly 5 weeks spec	397.	305.1	{267., 336.}	23.1486	0.115976	newly 5 weeks cumul	2702.	2366.87	{2230., 2483.}	12.403	0.0578551	newly 54 weeks cumul	4210.	3230.1	{3098., 3359.}	23.2755	0.115318
3	newly 3 weeks spec	586.	617.47	{579., 660.}	6.51365	0.0272741	newly 3 weeks cumul	2929.	2947.87	{2866., 3009.}	1.65483	0.00716527	newly 5 weeks spec	256.	232.93	{203., 261.}	10.3477	0.0487399	newly 5 weeks cumul	3549.	3562.39	{3426., 3638.}	1.77036	0.00771128	newly 60 weeks cumul	3885.	3902.76	{3698., 3985.}	2.1668	0.00945511
4	newly 3 weeks spec	581.	470.49	{436., 509.}	19.0207	0.0924025	newly 3 weeks cumul	2166.	1830.05	{1759., 1892.}	15.5102	0.0734539	newly 5 weeks spec	320.	257.18	{229., 287.}	19.6875	0.0967154	newly 5 weeks cumul	2954.	2443.27	{2363., 2539.}	17.2894	0.0826796	newly 54 weeks cumul	3942.	3048.46	{2936., 3161.}	22.6672	0.111918
5	newly 3 weeks spec	669.	638.32	{595., 681.}	5.51868	0.0250268	newly 3 weeks cumul	2580.	2464.35	{2403., 2531.}	4.52907	0.0203214	newly 5 weeks spec	386.	357.88	{320., 401.}	9.33161	0.0432891	newly 5 weeks cumul	3457.	3303.61	{3186., 3413.}	4.56957	0.0205743	newly 57 weeks cumul	4587.	4212.76	{3964., 4467.}	8.15871	0.0377289
6	newly 3 weeks spec	808.	712.17	{628., 774.}	11.9121	0.056429	newly 3 weeks cumul	2954.	2810.9	{2641., 2920.}	4.86865	0.0220139	newly 5 weeks spec	362.	367.09	{288., 423.}	13.0028	0.0570434	newly 5 weeks cumul	3891.	3695.9	{3385., 3879.}	5.07633	0.0232661	newly 58 weeks cumul	4944.	4517.15	{3957., 4851.}	8.6337	0.040812
7	newly 3 weeks spec	755.	663.	{108., 902.}	32.9828	0.251684	newly 3 weeks cumul	2728.	2592.58	{1160., 3171.}	25.6349	0.142056	newly 5 weeks spec	406.	366.2	{16., 526.}	43.8177	0.521121	newly 5 weeks cumul	3682.	3451.39	{1204., 4340.}	29.7629	0.175185	newly 59 weeks cumul	4694.	4383.35	{1259., 5647.}	33.9329	0.210728
8	newly 3 weeks spec	735.	616.	{546., 724.}	16.9959	0.0824707	newly 3 weeks cumul	2632.	2363.06	{2196., 2731.}	11.7257	0.0544048	newly 5 weeks spec	475.	325.59	{280., 411.}	31.4547	0.167758	newly 5 weeks cumul	3719.	3142.99	{2933., 3711.}	15.8163	0.0760144	newly 56 weeks cumul	5155.	3860.19	{3567., 4799.}	25.1176	0.127984
9	newly 3 weeks spec	675.	701.48	{666., 736.}	4.54519	0.0191132	newly 3 weeks cumul	2453.	2507.88	{2466., 2551.}	2.37016	0.0101495	newly 5 weeks spec	427.	440.78	{413., 464.}	4.62295	0.0195295	newly 5 weeks cumul	3435.	3516.77	{3456., 3563.}	2.42358	0.0103736	newly 56 weeks cumul	4640.	4906.1	{4860., 4964.}	5.74698	0.0242336
10	newly 3 weeks spec	697.	683.28	{624., 736.}	5.13917	0.022872	newly 3 weeks cumul	2508.	2350.98	{2264., 2426.}	6.26077	0.0282164	newly 5 weeks spec	467.	450.09	{353., 510.}	9.55246	0.0446401	newly 5 weeks cumul	3582.	3366.63	{3139., 3492.}	6.01591	0.0273288	newly 56 weeks cumul	5280.	4829.8	{4083., 5160.}	8.52652	0.0407172
11	newly 3 weeks spec	649.	291.43	{198., 441.}	55.0955	0.366259	newly 3 weeks cumul	2299.	1450.89	{1185., 1810.}	36.8904	0.205026	newly 5 weeks spec	447.	107.57	{55., 207.}	75.9351	0.677851	newly 5 weeks cumul	3330.	1732.68	{1365., 2334.}	47.9676	0.291995	newly 51 weeks cumul	4840.	1883.56	{1437., 2722.}	61.0835	0.422131
12	newly 3 weeks spec	799.	757.03	{714., 801.}	5.67084	0.0256421	newly 3 weeks cumul	2755.	2756.84	{2682., 2823.}	1.81343	0.00792489	newly 5 weeks spec	494.	417.83	{385., 448.}	15.419	0.0734783	newly 5 weeks cumul	3861.	3747.24	{3674., 3833.}	3.0619	0.0136098	newly 56 weeks cumul	5044.	4685.28	{4583., 4777.}	7.11182	0.0321328
13	newly 3 weeks spec	580.	411.16	{353., 456.}	29.1103	0.151698	newly 3 weeks cumul	2137.	1698.34	{1521., 1828.}	20.5269	0.10079	newly 5 weeks spec	319.	205.72	{160., 245.}	35.511	0.196646	newly 5 weeks cumul	2908.	2198.96	{1932., 2399.}	24.3824	0.122818	newly 52 weeks cumul	3866.	2621.92	{2273., 2891.}	32.18	0.170678
14	newly 3 weeks spec	614.	418.98	{378., 452.}	32.5635	0.171943	newly 3 weeks cumul	2380.	1847.81	{1733., 1921.}	22.7508	0.1125	newly 5 weeks spec	361.	183.66	{153., 212.}	49.651	0.30226	newly 5 weeks cumul	3257.	2312.8	{2174., 2399.}	29.3528	0.151466	newly 57 weeks cumul	4210.	2631.59	{2449., 2736.}	38.005	0.208435
15	newly 3 weeks spec	533.	651.79	{615., 682.}	22.2871	0.0870378	newly 3 weeks cumul	2029.	2288.3	{2234., 2341.}	12.7797	0.0521531	newly 5 weeks spec	261.	421.89	{387., 452.}	61.6437	0.207826	newly 5 weeks cumul	2645.	3238.78	{3172., 3298.}	22.4491	0.0879107	newly 54 weeks cumul	3346.	4617.25	{4557., 4674.}	37.9931	0.139831
16	newly 3 weeks spec	754.	808.	{769., 843.}	7.28117	0.0303019	newly 3 weeks cumul	2774.	2945.35	{2893., 2998.}	6.177	0.0259851	newly 5 weeks spec	425.	475.85	{450., 500.}	12.0306	0.0489153	newly 5 weeks cumul	3788.	4054.69	{3992., 4114.}	7.04039	0.0295208	newly 57 weeks cumul	4918.	5369.44	{5330., 5411.}	9.17934	0.0381327
17	newly 3 weeks spec	715.	477.53	{430., 522.}	33.3329	0.17892	newly 3 weeks cumul	2771.	2160.57	{2002., 2295.}	22.157	0.109695	newly 5 weeks spec	457.	186.24	{162., 215.}	59.2473	0.395798	newly 5 weeks cumul	3767.	2652.61	{2469., 2800.}	29.618	0.153765	newly 59 weeks cumul	4791.	2923.78	{2723., 3140.}	38.9735	0.216028
18	newly 3 weeks spec	721.	614.5	{469., 716.}	15.0985	0.0766652	newly 3 weeks cumul	2736.	2414.87	{2077., 2623.}	11.7372	0.0564168	newly 5 weeks spec	402.	331.44	{209., 415.}	19.1741	0.108311	newly 5 weeks cumul	3695.	3204.89	{2615., 3553.}	13.2641	0.0657455	newly 57 weeks cumul	4724.	4044.96	{3005., 4667.}	14.3743	0.0755723
19	newly 3 weeks spec	716.	849.81	{822., 892.}	20.9511	0.0838558	newly 3 weeks cumul	3184.	3366.67	{3335., 3456.}	7.54617	0.0323093	newly 5 weeks spec	379.	414.32	{391., 452.}	13.6887	0.0600627	newly 5 weeks cumul	4076.	4387.64	{4386., 4497.}	9.83857	0.0418855	newly 59 weeks cumul	4764.	5205.64	{5229., 5338.}	11.95	0.0507848
20	newly 3 weeks spec	765.	682.41	{556., 749.}	11.0157	0.0532515	newly 3 weeks cumul	2647.	2556.97	{2212., 2758.}	5.76162	0.0264259	newly 5 weeks spec	468.	373.17	{264., 441.}	20.3013	0.105222	newly 5 weeks cumul	3727.	3444.43	{2847., 3683.}	7.8908	0.0374444	newly 56 weeks cumul	5189.	4340.56	{3282., 4855.}	16.4463	0.0816321
21	newly 3 weeks spec	673.	578.87	{544., 613.}	13.9866	0.0659311	newly 3 weeks cumul	2256.	2085.71	{2023., 2142.}	7.54832	0.034183	newly 5 weeks spec	405.	352.19	{320., 375.}	13.0494	0.0616276	newly 5 weeks cumul	3166.	2896.86	{2817., 2966.}	8.50095	0.0386724	newly 53 weeks cumul	4383.	3890.74	{3798., 3969.}	11.2311	0.0517993
22	newly 3 weeks spec	723.	678.47	{589., 752.}	7.89073	0.036558	newly 3 weeks cumul	3471.	3228.18	{3104., 3366.}	6.99568	0.031729	newly 5 weeks spec	278.	240.66	{183., 288.}	15.5827	0.0772454	newly 5 weeks cumul	4204.	3877.68	{3603., 4067.}	7.76546	0.0355517	newly 60 weeks cumul	4711.	4207.68	{3816., 4468.}	10.6839	0.0498275
23	newly 3 weeks spec	587.	405.61	{249., 491.}	30.9012	0.174655	newly 3 weeks cumul	1900.	1601.08	{1186., 1824.}	15.7326	0.0791931	newly 5 weeks spec	330.	212.2	{90., 283.}	35.697	0.23084	newly 5 weeks cumul	2657.	2109.84	{1467., 2464.}	20.5932	0.108026	newly 55 weeks cumul	3467.	2576.6	{1656., 3082.}	25.6821	0.142196
24	newly 3 weeks spec	579.	584.72	{559., 612.}	2.86356	0.0123319	newly 3 weeks cumul	2054.	2142.16	{2091., 2183.}	4.30282	0.0182363	newly 5 weeks spec	382.	368.19	{343., 394.}	4.98691	0.0223705	newly 5 weeks cumul	2904.	2979.75	{2931., 3016.}	2.62156	0.0112143	newly 53 weeks cumul	4101.	4188.49	{4143., 4234.}	2.13338	0.00915398
25	newly 3 weeks spec	727.	453.2	{382., 591.}	37.6616	0.210763	newly 3 weeks cumul	3100.	2377.11	{2155., 2812.}	23.319	0.117376	newly 5 weeks spec	309.	145.2	{104., 213.}	53.0097	0.341497	newly 5 weeks cumul	3847.	2782.15	{2519., 3389.}	27.68	0.143373	newly 56 weeks cumul	4343.	2956.16	{2641., 3751.}	31.9328	0.170381
26	newly 3 weeks spec	523.	452.16	{385., 504.}	22.6577	0.105029	newly 3 weeks cumul	2145.	2155.35	{1947., 2265.}	7.95571	0.0326197	newly 5 weeks spec	188.	167.46	{118., 201.}	32.4894	0.143622	newly 5 weeks cumul	2653.	2597.53	{2338., 2718.}	11.5398	0.0479442	newly 59 weeks cumul	2904.	2846.52	{2496., 2866.}	14.9208	0.0605115
27	newly 3 weeks spec	757.	761.35	{610., 846.}	10.5033	0.0468164	newly 3 weeks cumul	2773.	2868.65	{2523., 3035.}	7.94555	0.0339702	newly 5 weeks spec	463.	425.29	{288., 501.}	14.2959	0.0733466	newly 5 weeks cumul	3879.	3871.96	{3258., 4150.}	8.62233	0.0385528	newly 57 weeks cumul	5146.	4946.2	{3777., 5442.}	11.0925	0.0529034
28	newly 3 weeks spec	747.	696.58	{604., 757.}	7.57697	0.0371593	newly 3 weeks cumul	3109.	3069.18	{2823., 3198.}	3.62303	0.0168415	newly 5 weeks spec	347.	293.08	{218., 334.}	15.879	0.0834892	newly 5 weeks cumul	3954.	3819.05	{3403., 3994.}	4.02656	0.0194991	newly 60 weeks cumul	4582.	4319.73	{3705., 4561.}	5.75404	0.0282909
29	newly 3 weeks spec	724.	552.67	{510., 590.}	23.6644	0.117908	newly 3 weeks cumul	2790.	2405.03	{2321., 2490.}	13.7982	0.0646265	newly 5 weeks spec	343.	231.43	{204., 256.}	32.5277	0.172313	newly 5 weeks cumul	3611.	2996.2	{2917., 3080.}	17.0258	0.0811689	newly 55 weeks cumul	4378.	3375.19	{3285., 3452.}	22.9057	0.113079
30	newly 3 weeks spec	685.	712.99	{681., 748.}	4.44526	0.0186956	newly 3 weeks cumul	2400.	2515.85	{2469., 2566.}	4.82708	0.020422	newly 5 weeks spec	366.	449.6	{420., 478.}	22.8415	0.0888358	newly 5 weeks cumul	3210.	3538.96	{3482., 3580.}	10.248	0.0423441	newly 55 weeks cumul	4078.	4920.4	{4857., 4970.}	20.6572	0.0815358
31	newly 3 weeks spec	731.	709.23	{682., 740.}	3.83721	0.0171678	newly 3 weeks cumul	2627.	2746.95	{2697., 2801.}	4.90255	0.0208373	newly 5 weeks spec	439.	375.09	{342., 403.}	14.5581	0.0693228	newly 5 weeks cumul	3631.	3645.59	{3601., 3702.}	1.22473	0.00541623	newly 58 weeks cumul	4700.	4519.77	{4447., 4645.}	3.84277	0.0172787
32	newly 3 weeks spec	583.	438.58	{399., 472.}	24.7719	0.124542	newly 3 weeks cumul	2074.	1720.98	{1632., 1788.}	17.0212	0.0813064	newly 5 weeks spec	399.	232.89	{205., 263.}	41.6316	0.235969	newly 5 weeks cumul	2998.	2281.72	{2187., 2387.}	23.8919	0.118798	newly 50 weeks cumul	4177.	2810.88	{2678., 2943.}	32.7058	0.172331
33	newly 3 weeks spec	525.	430.48	{379., 476.}	18.0076	0.0878045	newly 3 weeks cumul	2485.	2220.34	{2082., 2369.}	10.6503	0.0494769	newly 5 weeks spec	210.	147.73	{124., 169.}	29.6905	0.156443	newly 5 weeks cumul	3022.	2621.32	{2455., 2812.}	13.2588	0.0624245	newly 58 weeks cumul	3330.	2818.59	{2623., 3037.}	15.3577	0.0731674
34	newly 3 weeks spec	536.	622.74	{592., 658.}	16.1828	0.0646766	newly 3 weeks cumul	2500.	2742.85	{2682., 2797.}	9.714	0.0402026	newly 5 weeks spec	238.	257.3	{236., 278.}	8.85714	0.0362755	newly 5 weeks cumul	3124.	3407.57	{3338., 3460.}	9.07714	0.0376862	newly 60 weeks cumul	3558.	3840.52	{3775., 3906.}	7.94042	0.0331409
35	newly 3 weeks spec	625.	588.64	{554., 624.}	6.1152	0.0277774	newly 3 weeks cumul	2116.	2095.7	{2039., 2154.}	1.86578	0.0081902	newly 5 weeks spec	382.	372.96	{345., 407.}	5.43979	0.0240799	newly 5 weeks cumul	2967.	2944.7	{2865., 3024.}	1.63667	0.00717236	newly 55 weeks cumul	4257.	4046.62	{3950., 4128.}	4.94198	0.022071
36	newly 3 weeks spec	695.	629.2	{595., 664.}	9.46763	0.0436182	newly 3 weeks cumul	2481.	2338.03	{2285., 2393.}	5.7626	0.025851	newly 5 weeks spec	436.	363.78	{337., 388.}	16.5642	0.0794274	newly 5 weeks cumul	3487.	3189.53	{3123., 3242.}	8.53083	0.0387773	newly 53 weeks cumul	5127.	4145.81	{4059., 4207.}	19.1377	0.0923053
37	newly 3 weeks spec	692.	594.59	{436., 667.}	14.1806	0.0716923	newly 3 weeks cumul	2700.	2484.78	{2041., 2643.}	7.9837	0.0383473	newly 5 weeks spec	388.	273.92	{159., 320.}	29.4639	0.165818	newly 5 weeks cumul	3670.	3170.01	{2476., 3408.}	13.6237	0.0669978	newly 56 weeks cumul	4686.	3682.28	{2727., 3984.}	21.4195	0.109641
38	newly 3 weeks spec	526.	405.14	{358., 442.}	22.9772	0.114855	newly 3 weeks cumul	2700.	2408.15	{2313., 2493.}	10.8093	0.0498904	newly 5 weeks spec	147.	106.44	{87., 126.}	27.5918	0.144743	newly 5 weeks cumul	3166.	2726.94	{2592., 2824.}	13.868	0.065092	newly 59 weeks cumul	3369.	2833.06	{2704., 2942.}	15.908	0.0755288
39	newly 3 weeks spec	662.	600.05	{549., 642.}	9.42447	0.0446643	newly 3 weeks cumul	2825.	2764.85	{2687., 2845.}	2.47257	0.0113567	newly 5 weeks spec	284.	243.65	{211., 272.}	14.2077	0.0710314	newly 5 weeks cumul	3546.	3397.37	{3245., 3503.}	4.19994	0.0194235	newly 59 weeks cumul	4001.	3796.32	{3556., 3928.}	5.11572	0.0239299
40	newly 3 weeks spec	671.	619.95	{587., 649.}	7.66766	0.0349659	newly 3 weeks cumul	2384.	2383.17	{2324., 2433.}	1.31753	0.00572335	newly 5 weeks spec	421.	330.51	{301., 356.}	21.4941	0.10592	newly 5 weeks cumul	3361.	3171.22	{3110., 3246.}	5.64653	0.0252996	newly 56 weeks cumul	4464.	3935.17	{3852., 4016.}	11.8466	0.0548175
41	newly 3 weeks spec	690.	477.6	{445., 514.}	30.7826	0.160476	newly 3 weeks cumul	2492.	2069.24	{1979., 2157.}	16.9647	0.0809582	newly 5 weeks spec	409.	212.73	{189., 240.}	47.9878	0.285831	newly 5 weeks cumul	3447.	2607.15	{2511., 2699.}	24.3647	0.121456	newly 55 weeks cumul	4485.	2976.97	{2877., 3082.}	33.6239	0.178156
42	newly 3 weeks spec	566.	360.49	{319., 407.}	36.3092	0.19793	newly 3 weeks cumul	1986.	1514.12	{1436., 1587.}	23.7603	0.118205	newly 5 weeks spec	394.	173.6	{137., 207.}	55.9391	0.361407	newly 5 weeks cumul	2865.	1938.56	{1797., 2073.}	32.3365	0.170256	newly 50 weeks cumul	4248.	2292.	{2061., 2493.}	46.0452	0.269119
43	newly 3 weeks spec	779.	804.21	{765., 846.}	5.63158	0.0243865	newly 3 weeks cumul	3579.	3590.04	{3541., 3691.}	2.19838	0.00978497	newly 5 weeks spec	296.	314.36	{283., 345.}	10.6757	0.0465133	newly 5 weeks cumul	4369.	4413.49	{4371., 4527.}	2.9794	0.0132771	newly 60 weeks cumul	4830.	4892.09	{4870., 5017.}	3.56708	0.0159974
44	newly 3 weeks spec	659.	479.	{417., 596.}	27.3141	0.141844	newly 3 weeks cumul	2196.	1826.19	{1677., 2148.}	16.964	0.0823614	newly 5 weeks spec	414.	272.51	{221., 356.}	34.1812	0.188508	newly 5 weeks cumul	3184.	2463.47	{2259., 3017.}	22.6297	0.113785	newly 51 weeks cumul	4755.	3120.73	{2770., 4085.}	34.3695	0.187058
45	newly 3 weeks spec	602.	629.31	{598., 659.}	5.29734	0.022324	newly 3 weeks cumul	2275.	2302.66	{2249., 2357.}	1.94901	0.00842342	newly 5 weeks spec	418.	378.11	{351., 403.}	9.70574	0.0450221	newly 5 weeks cumul	3189.	3174.35	{3110., 3237.}	1.35748	0.00598265	newly 55 weeks cumul	4496.	4241.27	{4176., 4334.}	5.6657	0.0254966
46	newly 3 weeks spec	631.	688.81	{661., 723.}	11.2441	0.0471211	newly 3 weeks cumul	2297.	2429.25	{2397., 2490.}	7.58424	0.0324746	newly 5 weeks spec	464.	441.81	{414., 473.}	6.03233	0.0296325	newly 5 weeks cumul	3301.	3429.35	{3412., 3518.}	6.15662	0.0270318	newly 53 weeks cumul	4939.	4861.2	{4892., 4996.}	2.30856	0.0122018
47	newly 3 weeks spec	796.	598.18	{548., 638.}	24.8518	0.124737	newly 3 weeks cumul	2859.	2461.88	{2374., 2550.}	13.8902	0.065118	newly 5 weeks spec	468.	274.04	{241., 308.}	41.4444	0.233983	newly 5 weeks cumul	3902.	3145.47	{3049., 3258.}	19.3883	0.0937475	newly 55 weeks cumul	5202.	3631.56	{3527., 3749.}	30.1892	0.156203
48	newly 3 weeks spec	746.	736.22	{682., 790.}	4.51206	0.0198949	newly 3 weeks cumul	2942.	2894.86	{2715., 3007.}	2.87967	0.0127987	newly 5 weeks spec	435.	368.5	{322., 405.}	15.2874	0.0737734	newly 5 weeks cumul	3949.	3790.23	{3486., 3939.}	4.10762	0.0185709	newly 58 weeks cumul	4973.	4531.24	{4105., 4737.}	8.90167	0.0409948
49	newly 3 weeks spec	753.	802.75	{771., 834.}	6.67331	0.0278352	newly 3 weeks cumul	3170.	3229.77	{3171., 3283.}	1.99968	0.00856807	newly 5 weeks spec	359.	389.49	{364., 415.}	8.83844	0.0362837	newly 5 weeks cumul	4008.	4184.5	{4119., 4253.}	4.40369	0.0186861	newly 59 weeks cumul	4662.	4953.86	{4893., 5004.}	6.28657	0.0264585
50	newly 3 weeks spec	849.	795.54	{565., 868.}	7.5053	0.0371625	newly 3 weeks cumul	3183.	2954.09	{2678., 3077.}	7.19164	0.0330954	newly 5 weeks spec	465.	439.1	{228., 510.}	13.3419	0.0724567	newly 5 weeks cumul	4282.	3991.85	{3168., 4206.}	6.77604	0.0322705	newly 57 weeks cumul	5322.	5048.21	{3444., 5444.}	7.20349	0.0364485
